# Altered Protease–Activated Receptor-1 Expression and Signaling in a Malignant Pleural Mesothelioma Cell Line, NCI-H28, with Homozygous Deletion of the β-Catenin Gene

**DOI:** 10.1371/journal.pone.0111550

**Published:** 2014-11-03

**Authors:** Alessandra Fazzini, Vanessa D’Antongiovanni, Laura Giusti, Ylenia Da Valle, Federica Ciregia, Ilaria Piano, Antonella Caputo, Anna Maria D’Ursi, Claudia Gargini, Antonio Lucacchini, Maria Rosa Mazzoni

**Affiliations:** 1 Department of Pharmacy, University of Pisa, Pisa, Italy; 2 Department of Pharmacy, University of Salerno, Salerno, Italy; University of Illinois at Chicago, United States of America

## Abstract

Protease activated receptors (PARs) are G-protein coupled receptors that are activated by an unique proteolytic mechanism. These receptors play crucial roles in hemostasis and thrombosis but also in inflammation and vascular development. PARs have also been implicated in tumor progression, invasion and metastasis. In this study, we investigated expression and signaling of PAR_1_ in nonmalignant pleural mesothelial (Met-5A) and malignant pleural mesothelioma (NCI-H28) cells. We found that the expression level of PAR_1_ was markedly higher in NCI-H28 cells compared to Met-5A and human primary mesothelial cells. Other three malignant pleural mesothelioma cell lines, i.e. REN, Ist-Mes2, and Mero-14, did not show any significant PAR_1_ over-expression compared to Met-5A cell line. Thrombin and PAR_1_ activating peptides enhanced Met-5A and NCI-H28 cell proliferation but in NCI-H28 cells higher thrombin concentrations were required to obtain the same proliferation increase. Similarly, thrombin caused extracellular signal-regulated kinase 1/2 activation in both cell lines but NCI-H28 cells responded at higher agonist concentrations. We also determined that PAR_1_ signaling through G_q_ and G_12/13_ proteins is severely altered in NCI-H28 cells compared to Met-5A cells. On the contrary, PAR_1_ signaling through G_i_ proteins was persistently maintained in NCI-H28 cells. Furthermore, we demonstrated a reduction of cell surface PAR_1_ expression in NCI-H28 and malignant pleural mesothelioma REN cells. Thus, our results provide evidences for dysfunctional PAR_1_ signaling in NCI-H28 cells together with reduced plasma membrane localization. The role of PAR_1_ in mesothelioma progression is just emerging and our observations can promote further investigations focused on this G-protein coupled receptor.

## Introduction

Malignant mesothelioma (MM) is a relatively rare but highly aggressive neoplasm arising from mesothelial cells on the serosal surfaces of the pleural, peritoneal and pericardial cavities. Asbestos fiber exposure is widely accepted as the main cause with approximately 80% of cases being directly attributed to occupational exposure [1]. Although asbestos exposure has a pivotal role in initiating both cellular and molecular events which lead to MM development other factors such as genetic and epigenetic alterations contribute to its pathogenesis [1]. Several growth factors and their target receptors have been implicated in the oncogenesis, progression and resistance to therapy of MM [1]. In addition, the chemokine CXL12 and its target receptor CXCR4 which belongs to the large family of seven-transmembrane G-protein coupled receptors (GPCRs), have been found to be highly expressed in malignant pleural mesothelioma (MPM) cell lines and tumor tissues suggesting they can be involved in tumor progression and survival [2].

Numerous evidences link aberrant GPCR expression and activation to several types of human malignancies [3], [4]. Among GPCRs, PARs are a subset which have a unique mechanism of activation. In fact, they are activated enzymatically through proteolysis by enzymes of the serine protease family [5]. The proteolytic cleavage occurs at specific sites within their N-terminal region, thereby exposing novel N-termini, and the ‘tethered ligand’ then folds back onto the extracellular loop II of the receptor, resulting in activation. There are four PARs encoded by distinct genes in the mammalian genome. The prototype of this GPCR subfamily is PAR_1_ which transmits cellular response to thrombin [6], [7]. The receptor subfamily also includes PAR_2_ which is activated by trypsin, and two other thrombin-activated receptors, PAR_3_ and PAR_4_ [8]–[10]. Other proteases besides trypsin for PAR_2_ and thrombin and trypsin for PAR_1_ and PAR_4_ can activate these receptors [11]. Additionally, synthetic peptides that mimic the first six amino acids of the newly formed N-terminus can act as soluble ligands in the absence of receptor proteolysis. Activated PAR_1_ couples to multiple heterotrimeric G-protein subtypes including G_i_, G_q_ and G_12/13_ [11], [12].

PARs have multiple roles in many physiological and pathological events involving different tissues and organs such as the cardiovascular, musculoskeletal, gastrointestinal, respiratory and central nervous system [13]. Coagulant proteases and PARs have been implicated in several types of malignant cancer. PAR_1_ is over-expressed in aggressive melanoma, colon cancer, prostate cancer, and invasive breast cancer [14]–[17], promoting tumor cell invasion and epithelial cell malignancy [17]–[20]. In addition, several proteases, which can activate PAR_1_ have been identified in tumors including tissue-derived trypsins, members of the coagulation cascade and matrix metalloprotease-1 [13], [21]. Finally, a recent study have shown that MPM cell lines that express tissue factor and PAR_1_ but not PAR_2_ are able to generate large tumors in nude mouse throracic cavities [22].

In the present study, we analyzed PAR_1_ expression levels, signaling and mitogenic effects in immortalized nonmalignant pleural mesothelial (Met-5A) and MPM cells (NCI-H28). In this MPM cell line, a homozygous deletion of the β-catenin gene (*CTNNB1*) has been demonstrated while thrombomodulin, a natural anticoagulant, appears to be silenced by an epigenetic mechanism [23], [24]. Therefore, we were interested to study PAR_1_ expression and signaling in this cell line and correlate our findings to known genetic and epigenetic alterations. Our work indicates that the expression levels of both PAR_1_ mRNA and protein are increased in NCI-H28 cells compared to those found in Met-5A and primary human mesothelial cells. In addition, the increased PAR_1_ expression appears to be an unique feature of the NCI-H28 cell line since in other three MPM cell lines, i.e. REN, Mero-14 and Ist-Mes2, PAR_1_ levels are not significantly different from that found in Met-5A cells. Perhaps more important, PAR_1_ signaling to down-stream effectors is dysfunctional as the signaling pathway through G_i_ is the only one that is fully maintained while G_12/13_ and G_q_ pathways are reduced. Furthermore, the mitogenic effect triggered by PAR_1_ activation is modified in NCI-H28 cells as compared to Met-5A cells. We also show that in this MPM cell line, cell surface PAR_1_ expression is reduced and the receptor mainly localizes in the intracellular compartment. The intracellular retention of PAR_1_ is likely responsible of the altered signaling.

## Materials and Methods

### Materials

Penicillin, streptomycin, hydrocortisone, cAMP, 4-(3-Butoxy-4-methoxybenzyl)-2-imidazolidinone, protease inhibitor cocktail, isoproterenol and secondary antibodies were products of Sigma-Aldrich Inc. (St. Louis, MO, USA). [^3^H]-cAMP (specific activity 31.0 Ci/mmol) and enhanced chemiluminescence substrate (Western lightning Plus-ECL) were from PerkinElmer Inc. (Waltham, MA, USA). The human microvascular endothelial cell (HMEC-1) line [25] was a generous gift of E.W. Ades (Centers for Disease Control, Atlanta, GA, USA) while NCI-H28 and Met-5A cells were purchased from LGC Standards s.r.l. (Middlesex, UK). REN mesothelioma cells [26], [27] were a generous gift of L. Moro (University of Piemonte Orientale “A. Avogadro”, Italy) while Mero-14 [28] and Ist-Mes2 [29] mesothelioma cells were kindly donated by Istituto Nazionale per la Ricerca sul Cancro (IST) - Genova (Italy). Mero-14, Ist-Mes2 and REN cells were verified for their identity by analyzing 10 to 18 genetic markers. Human adult primary mesothelial cells and their growth medium (MSO-1) were purchased from Zen-Bio, Inc (Research Triangle Park, NC, USA). Medium 199, MCDB-131 medium, RPMI-1640, DMEM, fetal bovine serum (FBS), trypsin-EDTA, epidermal growth factor (EGF), L-glutamine, human recombinant insulin, nitrocellulose membrane, Lipofectamine 2000 transfection reagent, Fluo-3 acetoxy methylester (Fluo-3 AM), pluronic acid, Alexa Fluor 488- and Alexa Fluor 568-labeled goat anti-mouse IgG and anti-rabbit IgG antibodies were purchased from Life Technologies Corporation (Carlsbad, CA, USA). Halt phoshatase inhibitor cocktail and 2,2′-azinobis (3-ethylbenzthiazoline-6-sulfonic acid) diammonium salt (1 step) were from Thermo Scientific (Waltham, MA, USA). WST-1 was a product of La Roche (Basel, Switzerland). RhoA activation assay kit was obtained from Cytoskeleton, Inc. (Denver, CO, USA). The PAR_1_ antagonist, SCH 79797 and the selective PAR_1_-activating peptide (PAR_1_-AP) (TFLLR-NH_2_) were products of Tocris Bioscience (Bristol, UK). The non-selective PAR_1_-AP (SFLLRN-NH_2_) was synthesized in Dr. A.M. D’Ursi’s laboratory (Department of Pharmacy, University of Salerno, Fisciano, Italy) using a conventional solid-phase strategy based on the Fmoc/t-Bu protection chemistry as previously described [30]. Human α-thrombin (high activity, ≥2,800 NIH U/mg protein) was a product of Calbiochem (EMD Millipore Biosciences, Billerica, MA, USA). The RNeasy Mini Kit and SYBR Green PCR Kit were purchased from Qiagen GMbH (Hilden, Germany). The Rev Transcription Kit was a product of New England BioLabs (Ipswich, MA, USA). A polyclonal anti-PAR_1_ antibody was from Santa Cruz Biotechnology Inc. (Santa Cruz, CA, USA) while a monoclonal anti-PAR_1_ antibody was from Abnova (Taipei City, Taiwan). A rabbit polyclonal anti-PAR_1_ antibody generated against the N-terminal sequence YEPFWEDEEKNESGLTEYC was a generous gift of Dr. J. Trejo (Department of Pharmacology, University of California, San Diego, CA, USA) [31]. Polyclonal anti-β-catenin, anti-caveolin-1, anti-phospho-p44/42 MAPK (extracellular signal-regulated kinase 1/2; ERK1/2), and anti-p44/42 MAPK (ERK1/2) antibodies were obtained from Cell Signaling Technology, Inc. (Danvers, MA, USA). A monoclonal anti-β-catenin antibody was from BD Biosciences (San Jose, CA, USA). A monoclonal anti-β-actin antibody was purchased from EMD Millipore Biosciences (Billerica, MA, USA). A vector containing the human β-catenin cDNA (pCMV6XL5-β-catenin-1), the pCMV6XL5 vector, a small interfering RNA (siRNA) directed against β-catenin, and a scrambled non-targeting siRNA (control siRNA) were purchased from OriGene (Rockville, MD, USA). Other agents and reagents were from standard commercial sources and were of the highest grade available.

### Cell culture

Met-5A cells were grown in Medium 199 supplemented with 10% FBS, 1% penicillin/streptomycin (100 units/ml/100 µg/ml), hydrocortisone (400 nM), EGF (3.3 nM) and human recombinant insulin (870 nM). NCI-H28 and REN cells were cultured in RPMI-1640 medium supplemented with 10% FBS and 1% penicillin/streptomycin (100 units/ml/100 µg/ml). Ist-Mes2 and Mero-14 cells were grown DMEM medium containing 4.5 g/ml glucose and 3.97 mM L-glutamine supplemented with 10% FBS and 1% penicillin/streptomycin (100 units/ml/100 µg/ml). Cells were normally propagated in their own growth media except before experiments they were plated in RPMI-1640 medium. Primary mesothelial cells were cultured in MSO-1 medium (Medium 199 supplemented with FBS, EGF, penicillin, streptomycin, amphotericin B) according to manufacturer’s instructions. HMEC-1 cells were grown as previously described [32]. All cells were cultured at 37°C and 5% CO_2_ in humidified atmosphere.

### Real time RT-PCR

RNA was isolated using the RNeasy Mini Kit (Qiagen) and tested for integrity by gel electrophoresis. mRNA was reverse transcribed to cDNA using a specific Rev Transcription Kit (New England BioLabs). Real time SYBR Green polymerase chain reaction (PCR) for PAR_1_ was performed using forward primer: 5′-TGCTTCAGTCTGTGCGG-3′; and reverse primer: 5′-CTCCATCAATAAAAGCAGTCCTCT-3′. The relative expression of PAR_1_, with β-actin as the reference gene, was determined using the MiniOpticon Real-Time PCR Detection System (BioRad Laboratories, Inc., Hercules, CA, USA). Data are presented as expression ratios normalized to β-actin.

### Western blot analysis

Human primary mesothelial cells grown in MSO-1 medium, Met-5A, NCI-H28, REN, Mero-14, and IstMes2 cells cultured in complete RPMI-1640 medium until confluence were washed with ice-cold PBS (8.1 mM Na_2_HPO_4_, 1.5 mM KH_2_PO_4_, pH 7.4, 137 mM NaCl and 2.7 mM KCl) and lysed in modified RIPA buffer (PBS, pH 7.4, 1% Igepal, 0.5% sodium deoxycholate, 0.1% SDS and 10 µl/ml protease inhibitor cocktail). Lysed cells were centrifuged at 14,000 g at 4°C for 45 min and supernatant was collected. To measure the protein content, the Bio-Rad DC protein assay kit (Bio-Rad Laboratories, Inc., Hercules, CA) was used with bovine serum albumin (BSA) as standard. Solubilized proteins (30 µg) were separated by 12% SDS-PAGE and transferred onto nitrocellulose. Immunoblots were carried out using a standard method as previously described [32], [33]. The immunoblot signal was visualized by using enhanced chemiluminescence substrate detection system (Western Lightning Plus-ECL). The chemiluminescent images were acquired by LAS4010 (GE Healthcare Life-Sciences, Pittsburgh, PA, USA). Intensity of immunoreactive bands was measured by densitometric scanning using Image Quant TL 1D, Version 7.0 (GE Healthcare Life-Sciences, Pittsburgh, PA, USA). Nitrocellulose membrane probed with anti-PAR_1_ antibodies was subsequently stripped and reprobed with the anti-β-actin antibody.

ERK1/2 activity was determined from 18 h serum and growth factor starved cells plated at 3×10^5^ density in 6-well dishes. After stimulation with different thrombin concentrations for 5 min, cells were lysed in modified TBS (50 mM Tris, pH 7.4, 150 mM NaCl, 0.1% Igepal, 10% glycerol, 1% protease and phosphatase inhibitor cocktails) and processed as described above. Activated ERK1/2 was detected by immunoblotting with anti-phospho-p44/42 MAPK (ERK1/2) antibody. Membranes were stripped and reprobed with anti-p44/42 MAPK (ERK1/2) antibody.

### Immunocytochemistry

NCI-H28 and Met-5A cells were seeded at 3×10^4^ cells per well in chamber slide (BD Biosciences, San Jose, CA, USA). Twenty-four hours later, cells were fixed in 2% paraformaldheyde in 0.1 M phosphate buffer, washed three times with PBS, rinsed, and blocked for 45 min with PBS containing 0.1% Triton-X 100 and 1% BSA. After washing, cells were incubated with mouse monoclonal anti-PAR_1_ (1∶100), mouse monoclonal anti-β-catenin (1∶500) or rabbit polyclonal anti-β-catenin (1∶1000) and rabbit polyclonal anti-caveolin-1 (1∶400) primary antibodies diluted in PBS containing 0.03% Triton-X 100 and 1% BSA for 18 h at 4°C. Double labelling studies were carried out as follow: anti-PAR_1_ and anti-caveolin-1; anti-PAR_1_ and rabbit polyclonal anti-β-catenin; mouse monoclonal anti-β-catenin and anti-caveolin-1. After washing, to visualize single staining, cells were incubated with Alexa Fluor 488- and Alexa Fluor 568-labeled goat anti-mouse (1∶400) or anti-rabbit (1∶400) antibodies for 2 hour at room temperature. Then slides were covered with Vectashield (Vector Laboratories, Burlingame, CA, USA). Confocal images were obtained with a Leica TCS-SP5 confocal microscope, using a 40×oil objective with 1.45 NA and a recommended pinhole size of less than 1.0 micrometer. The images were processed with PhotoshopCS3 software. To evaluate fluorescence colocalization, the images were also analyzed using the freely available ImageJ program [34].

### Cell proliferation assay

Met-5A and NCI-H28 cells were plated at 3×10^3^ cells/well in clear 96-well dishes and allowed to adhere overnight. Then cells were serum and growth factor starved for 12 hours and stimulated with and without agonists for 72 hours. After that, 10 µl of WST-1 mixture was added to each well, mixed gently for one min and cells incubated for additionally 2 hours at 37°C. Finally, the formazan dye was quantified by measuring the absorbance of each sample against background as blank with a Wallac 1420 multilabel counter microplate reader (PerkinElmer, Inc., Boston, MA, USA) at a wavelength of 450 nm.

### [Ca^2+^]_i_ measurement

PAR-induced increase of [Ca^2+^]_i_ was assessed by measuring fluorescence variations after agonist stimulation of cells loaded with Fluo-3 AM using a Wallac 1420 multilabel counter microplate reader (PerkinElmer, Inc., Boston, MA, USA), as previously described [32]. Cells were seeded in black/clear bottom 96-well assay plates at a density of 2×10^4^ cells/well (HMEC-1) or 1.5×10^4^ cells/well (Met-5A and NCI-H28) in complete growth media. After attachment, cells were starved in serum and growth factor free media containing BSA for 3 h at 37°C. Before starting the assay, cells were washed twice with loading buffer (20 mM Hepes, 0.83 mM Na_2_HPO_4_, 0.17 mM NaH_2_PO4, pH 7.4, 130 mM NaCl, 5 mM KCl, 2 mM CaCl_2_, and 1 mM MgSO_4_) containing 25 mM mannose, 1 mg/ml BSA and 2.5 mM probenecid and then incubated in 100 µl of the same buffer containing 6 µM Fluo-3 AM/0.024% pluronic acid. After 1 h at 37°C, cells were washed twice with loading buffer and incubated in 100 µl of the same buffer for an additional 1 h at 37°C. Fluorescence was recorded at baseline and every 3 seconds after thrombin (10 nM) or PAR_1_-APs (10 µM) addition for another 120 seconds.

### RhoA activation assay

Levels of GTP-bound RhoA were determined in serum and growth factor starved (18 h) Met-5A and NCI-H28 cells before and 2 min after stimulation with 10 nM thrombin or 10 µM selective PAR_1_-AP using a G-LISA RhoA activation assay kit (Cytoskeleton, Denver, CO, USA).

### Measurement of intracellular cAMP

Intracellular cAMP levels were measured using a competitive protein binding method as previously described [32]. Met-5A and NCI-H28 cells (4×10^4^/well) were plated in 24-well dishes and allowed to grow for 24 h. Thereafter, cells were incubated for 15 min in serum and growth factor free media containing 20 µM 4-(3-Butoxy-4-methoxybenzyl)-2-imidazolidinone and then exposed to different thrombin or selective PAR_1_-AP concentrations in the presence and absence of 100 nM SCH 79797 for 15 min. Assays were initiated by the addition of 1 µM isoproterenol.

### Cell surface ELISA

Detection of endogenous PAR_1_ expressed on the cell surface was quantified by ELISA essentially as described by Paing *et al.* [35]. Met-5A and NCI-H28 cells were plated in 24-well dishes at 6×10^4^ cell/well and grown overnight. Assay media was RPMI-1640 supplemented with 1 mg/ml BSA and 1% penicillin/streptomycin. Cells were washed with media and incubated on ice for 30 min. Afterwards, cells were washed and incubated with 5 µM SCH 79797 for 30 min and then treated with 10 nM thrombin for 10 min at 37°C. Cells were then washed with PBS and fixed with 4% paraformaldehyde on ice for 10 min. After fixation, cells were washed with PBS and incubated with primary antobody for 1 h at room temperature, followed by incubation with horseradish peroxidase-conjugate goat anti-rabbit secondary antibody for 1 h at room temperature. After washing, cells were incubated with horseradish peroxidase substrate 1 step 2,2′-azinobis (3-ethylbenzthiazoline-6-sulfonic acid) diammonium salt at room temperature. An aliquot was removed from each well and optical density was determined at 405 nm using a Wallac 1420 multilabel counter microplate reader.

### Transient β-catenin transfection and RNA interference

NCI-H28 and Met-5A cells were seeded onto 24-well plates at 5×10^4^ cells/well and transfected 24 h later with 0.7 µg/well pCMV6XL5-β-catenin or empty vector and 30 nM β-catenin or scrambled non-targeting siRNAs, respectively. Transfections were carried out for 48 h in RPMI-1640 using Lipofectamine 2000 according to the manufacturer’s suggested conditions (Invitrogen). ELISA assays for detection of cell surface PAR_1_ in transfected cells were performed as described above.

### Data analysis

Data analysis was performed by the computer program GraphPad Prism Version 4.0 for Windows (GraphPad Software, San Diego, CA, USA). Values represent the means ± S.E.M. of at least three independent experiments. The statistical significance of value differences was evaluated by one-way ANOVA followed by Bonferroni’s multiple comparison test using GraphPad Prism Version 4.0 for Windows. The Pearson’s correlation coefficient was used as statistic for quantifying fluorescence colocalization in confocal images.

## Results

PARs and their potential activating proteases are frequently over-expressed in human tumor tissues, including prostate cancer, invasive breast cancer, colon cancer, and malignant melanoma [14]–[20]. Lee *et al.* [36] have shown that PAR_2_ is present in human pleural tissues where it plays a role in pleural inflammatory responses while in primary cultures of human peritoneal mesothelial cells the expression of PAR_1_ has been reported [37]. In addition, the expression of PAR_1_ has been revealed in 3 MPM cell lines by western blot analysis but these cell lines do not express PAR_2_ [22]. Therefore, we decided to investigate expression and signaling of PAR_1_ in human pleural mesothelial and MPM cells to evaluate the possible role of this receptor in mesothelioma cell proliferation. For this work we utilized the MPM cell line, NCI-H28, which does not express CXCR4 and the nonmalignant pleural mesothelial cell line, Met-5A, was used as a control [2]. In this MPM cell line, apart from a homozygous deletion of the β-catenin gene (*CTNNB1*) a down-regulation of thrombomodulin expression by an epigenetic mechanism has been described [23], [24]. The expression of thrombomodulin, a glycosylated transmembrane protein which binds with high affinity to thrombin inhibiting its enzymatic activity and accelerating protein C activation, is lower in MPM tissue than in normal mesothelium [24]. In addition, low or no expression of thrombomodulin in various cancers has been associated with poor prognosis [38]–[40].

### PAR_1_ is over-expressed in NCI-H28 cells

**Figure 1:**
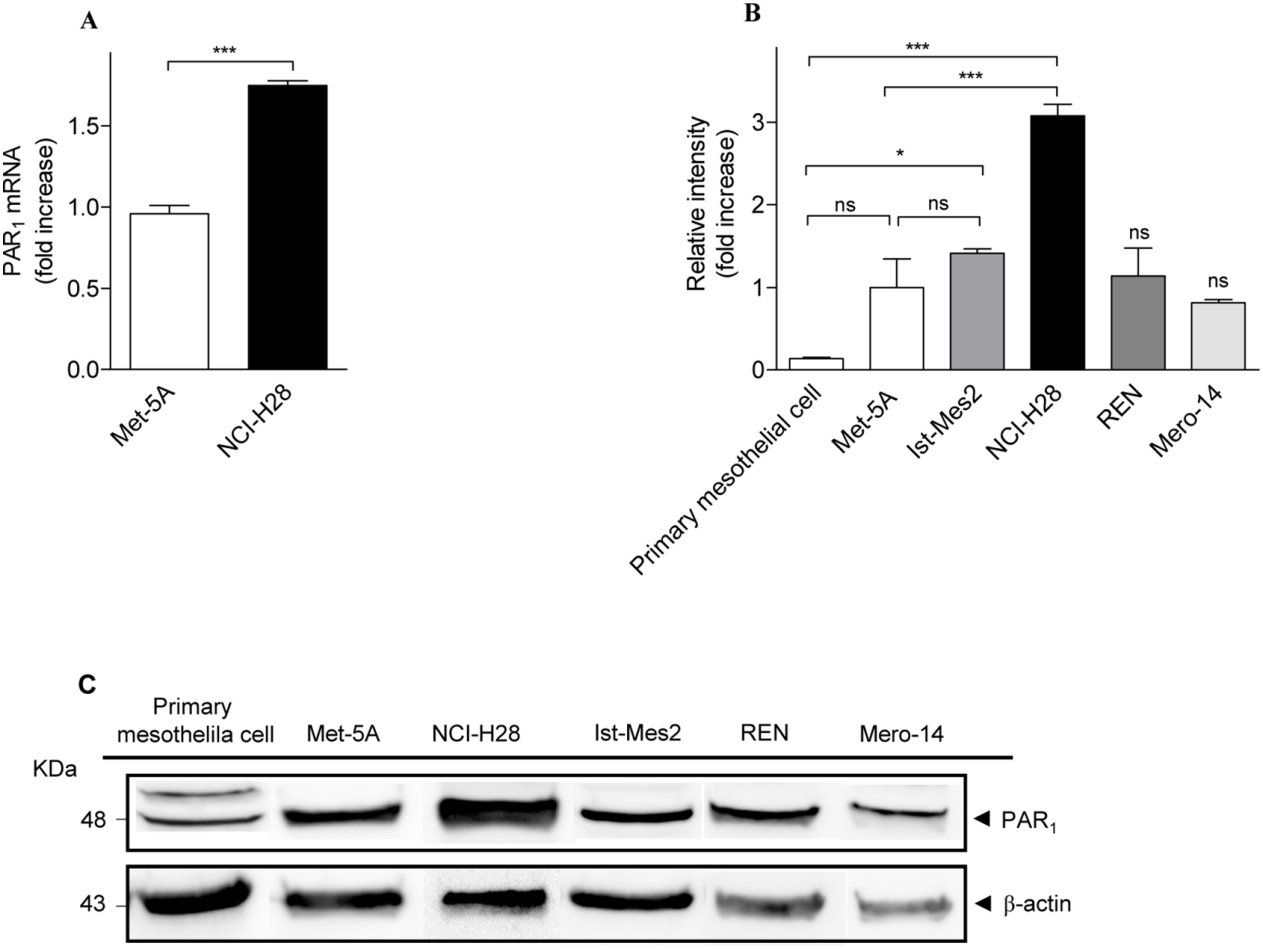
NCI-H28 cells over-express PAR_1_. A, relative expression levels of PAR_1_ mRNA in Met-5A and NCI-H28 cells as determined by real time RT-PCR. B, relative expression levels of PAR_1_ protein in primary mesothelial cells, Met-5A, NCI-H28, REN, Ist-Mes2, and Mero-14 cell lines as determined by immunoblot analysis followed by densitometric quantitation. Data are expressed as arbitrary unit (fold increase over Ctrl, Met-5A cells) after normalization by β-actin. Data shown are mean ± SEM of three independent experiments. The differences in PAR_1_ expression levels between Ctrl (Met-5A or primary mesothelial cells) and MPM cells were significant (*P≤0.05, ***P≤0.001) by one-way ANOVA followed by Bonferroni’s multiple comparison test (n = 3). C, a representative immunoblot.

To verify whether PAR_1_ mRNA level was different in malignant NCI-H28 cells compared to nonmalignant Met-5A cells, real time RT-PCR was performed using RNA extracted from these cells. In NCI-H28 cells, PAR_1_ mRNA level was significantly increased compared to Met-5A cells (Figure 1.A). Immunoblot analysis showed a 48 kDa band corresponding to PAR_1_ in lysates of Met-5A, NCI-H28 and other three MPM (Ist-Mes2, REN and Mero-14) cell lines while two close bands were detectable in immunoblot of human primary mesothelial cell lysates (Figure 1.C). The appearance of two bands was not a surprise since human PAR_1_ contains multiple glycosylation consensus sites and several studies have shown the detection of 40 to 100 kDa bands on immunoblots [41]–[43]. However, the PAR_1_ protein expression was lower in primary mesothelial cells than in Met-5A cells (Figure 1.B and 1.C). In NCI-H28 cells, the protein expression level was significantly increased compared to primary mesothelial and Met-5A cells (Figure 1.B and 1.C). In the other MPM cell lines, PAR_1_ protein levels were essentially similar to that found in Met-5A cells. Therefore, the increased PAR_1_ expression is an unique feature of NCI-H28 cell line. Overall, these findings suggest that the increased expression of PAR_1_ in NCI-H28 cells results from increased gene transcription although enhanced PAR_1_ mRNA and/or PAR_1_ protein stability can also be involved. We also examined PAR_2_ mRNA and protein levels in Met-5A and NCI-H28 cells (data not shown). Real time RT-PCR and western blot analysis demonstrated PAR_2_ expression levels were similar in both cell lines.

### PAR_1_ agonists enhance Met-5A and NCI-H28 cell proliferation

**Figure 2:**
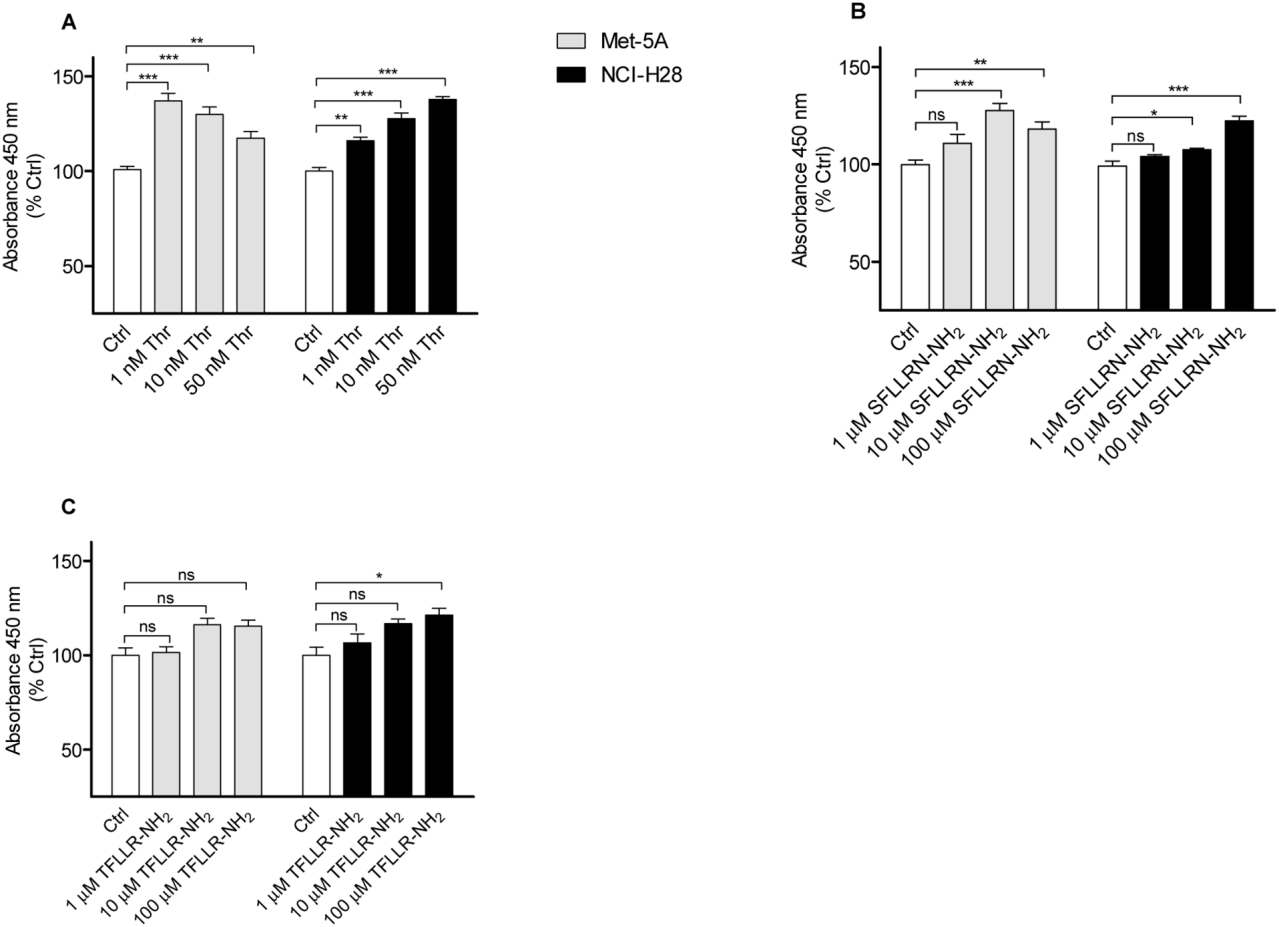
PAR_1_ agonists enhance Met-5A and NCI-H28 cell proliferation. Met-5A and NCI-H28 cells were serum and growth factor starved for 18 h and then treated with different concentrations of agonists for 72 h. Cell proliferation was measured using a mitochondrial activity assay (WST-1). The optical density values of vehicle treated Met-5A and NCI-H28 cells (Ctrls) were 0.210±0.03 and 0.232±0.04 (n = 6; ns by Student’s t test), respectively. A, thrombin-induced cell proliferation (n = 6); B, non-selective PAR_1_-AP-induced cell proliferation (n = 6); C, selective PAR_1_-AP-induced cell proliferation (n = 3). Data shown are mean ± SEM of at least three independent experiments performed in triplicate. The differences in proliferation between Ctrl and agonist-treated cells were significant (*P≤0.05, **P≤0.01, ***P≤0.001) by one-way ANOVA followed by Bonferroni’s multiple comparison test.

Next, we examined whether in NCI-H28 cells, PAR_1_ was functionally active by evaluating thrombin- or PAR_1_-APs-induced cell proliferation. Met-5A and NCI-H28 cells were incubated with various thrombin or PAR_1_-AP concentrations for 72 h. In Figure 2.A, the proliferative responses induced by thrombin stimulation are reported. Both Met-5A and NCI-H28 cells showed significant increases of cell proliferation at 72 h (Figure 2.A). However, the pattern of the proliferative response was quite different in NCI-H28 cells compared to that of Met-5A cells. As an example, in Met-5A the proliferative response was maximal at 1 nM thrombin with a progressive decrease up to 50 nM while in NCI-H28 cells the maximal response was reached at 50 nM (Figure 2.A).

The non-selective PAR_1_-AP, SFLLRN-NH_2_, was less effective than thrombin in stimulating Met-5A and NCI-H28 cell proliferation (Figure 2.B). A 24–28% increase of cell proliferation was reached at 10 and 100 µM SFLLRN-NH_2_ in Met-5A and NCI-H28 cells, respectively (Figure 2.B). The selective PAR_1_-AP, TFLLR-NH_2_, was less efficacious in stimulating cell proliferation than SFLLRN-NH_2_ but a concentration of 100 µM caused a 20% increase of NCI-H28 cell proliferation (Figure 2.C). These results highlight that PAR_1_-APs do not behave exactly as thrombin in stimulating cell proliferation.

### Reduced cell surface PAR_1_ expression in NCI-H28 cells

**Figure 3:**
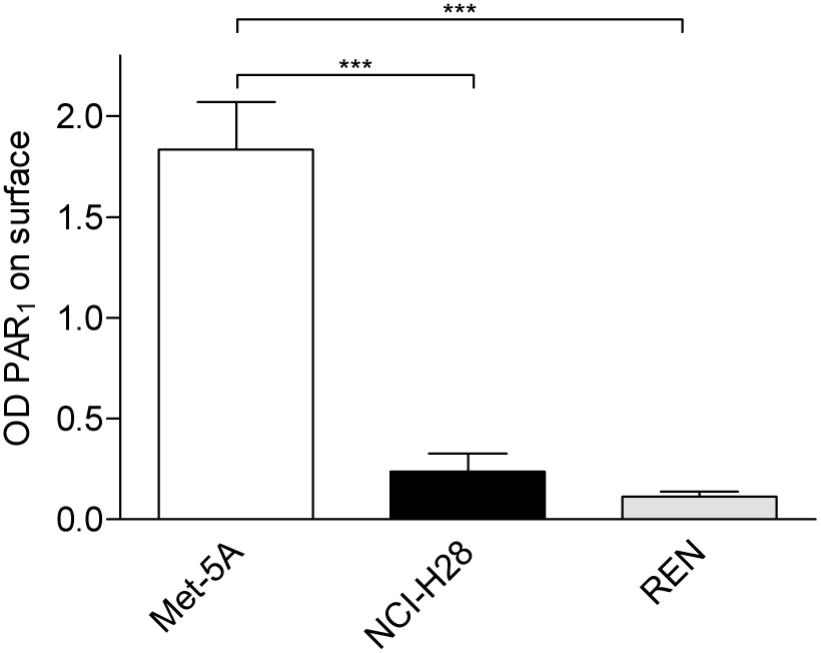
NCI-H28 and REN cells express significant less amount of PAR_1_ on plasma membrane. Cell surface PAR_1_ expression was measured by ELISA using a polyclonal antibody which recognizes the N-terminus of PAR_1_ [31]. Antibody binding to fixed cells was detected by horseradish peroxidise-conjugated secondary antibody. Data represent the mean ± SEM of three independent experiments performed in triplicate. The differences in cell surface PAR_1_ expression between Ctrl (Met-5A cells) and MPM (NCI-H28 and REN) cellswere significant (***P≤0.001) by one-way ANOVA followed by Bonferroni’s multiple comparison test.

Since NCI-H28 cells respond with proliferation at higher thrombin concentrations even though they express increased PAR_1_ levels (see Figure 1), we questioned whether the receptor is properly localized on cell surface in this cell line. Therefore, we compared the amount of cell surface PAR_1_ in Met-5A, NCI-H28 and REN cells using an ELISA assay. Interestingly, NCI-H28 cells showed significantly less cell surface PAR_1_ expression than Met-5A cells (Figure 3). REN cells, which express β-catenin as indicated by immunoblot analysis (data not shown), also showed a reduced cell surface receptor expression compared to Met-5A cells (Figure 3). Overall, these findings provide evidences of an altered cell surface distribution of PAR_1_ in two MPM cells lines showing different levels of total receptor expression.

### Dysfunctional PAR_1_ signaling in NCI-H28 cells

**Figure 4:**
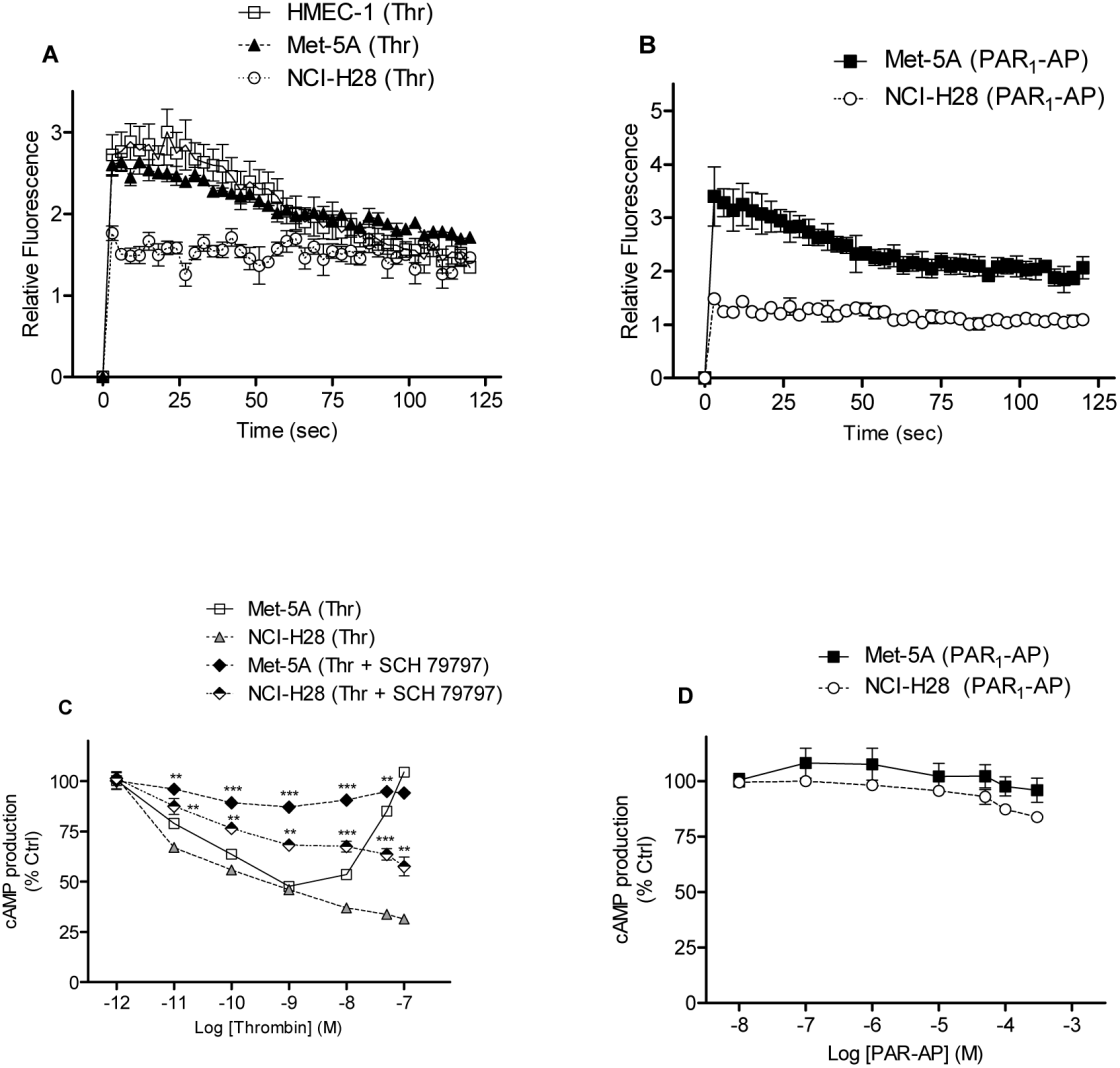
PAR_1_ agonist-induced G_q_ but not G_i_ signaling is impaired in NCI-H28 cells. A, thrombin-induced intracellular Ca^2+^ mobilization in HMEC-1, Met-5A, and NCI-H28 cells. B, selective-PAR1-AP-induced intracellular Ca^2+^ mobilization in Met-5A and NCI-H28 cells. Serum and growth factor starved cells were loaded with Fluo-3AM to measure [Ca^2+^]_i_ variations as indicated by changes in fluorescence intensity. Fluorescence was recorded before agonist addition (F_0_) and then every 3 seconds after thrombin (10 nM) or PAR_1_-AP (10 µM) addition for another 120 seconds. Data shown are mean ± SEM of a single experiment done in triplicate. Experiments were repeated two additional times with similar results. The results are reported as relative fluorescence (RF = F/F_0_ where F_0_ is basal fluorescence and F is fluorescence recorded after cell stimulation with the agonist). C, inhibition of isoproterenol stimulated cAMP production in Met-5A and NCI-H28 cells by different concentrations of thrombin in the presence and absence of 100 nM SCH 79797. D, no effect of the selective PAR_1_-AP on isoproterenol stimulated cAMP production in Met-5A and NCI-H28 cells. Serum and growth factor starved cells were exposed to different agonist concentrations. Assays were initiated by the addition of 1 µM isoproterenol. Production of cAMP was measured using a competition binding assay which includes the bovine adrenal cAMP binding protein and [^3^H]cAMP. Data shown are mean ± SEM of three independent experiments performed in triplicate. The differences between thrombin- and thrombin plus SCH 79797-treated cells were significant (**P≤0.01, ***P≤0.001) by one-way ANOVA followed by Bonferroni’s multiple comparison test (n = 3).

To further explore PAR_1_ ability of signaling in the NCI-H28 cell line, receptor-activated G_q_, G_i_, and G_12/13_ signaling pathways were examined. First, we investigated PAR_1_-activated G_q_ signaling by analyzing intracellular Ca^2+^ mobilization after cell stimulation with either thrombin or the selective PAR_1_-AP. As indicated by relative fluorescence increase, both thrombin (10 nM) and PAR_1_-AP (10 µM) induced rapid and transient increase of [Ca^2+^]_i_ in Met-5A as well as in HMEC-1 as previously reported (Figure 4.A and 4.B) [32]. In contrast, thrombin- or PAR_1_-AP-stimulation of NCI-H28 cells resulted in a reduced increase of [Ca^2+^]_i_ (Figure 4.A and 4.B). Given the sharply contrasting results, we examined both cell lines for the expression levels of some components of the G_q_ signaling pathway by immunoblot analysis (Figure S1). Whereas PLC-β_1_ was expressed at similar levels in both cell lines, the amount of Gα_q_ was apparently greater in NCI-H28 than Met-5A cells (Figure S1).

To explore the functional integrity of G_i_ signaling pathway, we analyzed thrombin- and PAR_1_-AP-induced inhibition of isoproterenol stimulated cAMP accumulation in both Met-5A and NCI-H28 cells. In Met-5A cells, 10 pM to 1 nM thrombin inhibited isoproterenol stimulated cAMP production in a concentration dependent manner reaching 50% inhibition at 1 nM (Figure 4.C). However, at higher thrombin concentrations (1 nM to 100 nM) the inhibitory effect was progressively diminished. In the presence of SCH 79797, the inhibitory effect of thrombin was reduced indicating that PAR_1_ mediates the effect. In NCI-H28 cells, thrombin inhibited cAMP in a concentration dependent manner reaching 50% and maximal inhibition (approximately 70%) at 1 nM and 100 nM, respectively (Figure 4.C). In the presence of SCH 79797, the inhibition curve was upwards shifted and the maximal inhibition at 100 nM was only 42% indicating that the inhibitory effect of cAMP accumulation is partially mediated by PAR_1_. Various concentrations of the selective PAR_1_-AP did not cause any inhibition of isoproterenol stimulated cAMP production in both Met-5A and NCI-H28 cells (Figure 4.D) demonstrating the functional selectivity of this peptide agonist.

**Figure 5:**
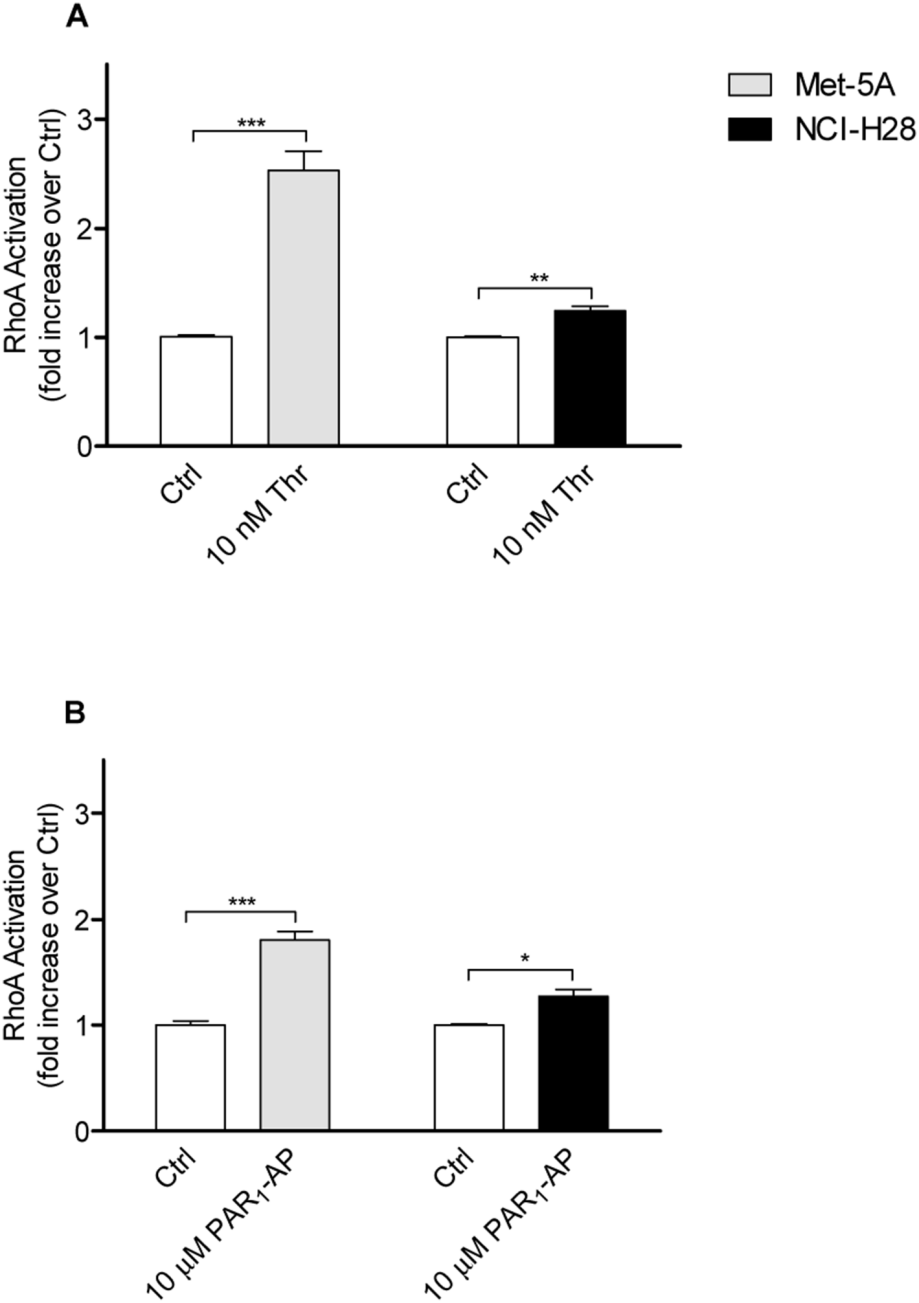
PAR_1_ agonist-induced G_12/13_ signaling is impaired in NCI-H28 cells. A, relative levels of RhoA activation in response to thrombin in Met-5A and NCI-H28 cells. B, relative levels of RhoA activation in response to the selective PAR_1_-AP in Met-5A and NCI-H28 cells. Rho A activation was measured in serum and growth factor starved cells using the RhoA G-LISA kit from Cytoskeleton. Data shown are mean ± SEM of three independent experiments performed in triplicate. The differences in RhoA activation between Ctrl (vehicle treated Met-5A or NCI-H28 cells) and agonist-treated cells were significant (*P≤0.05, **P≤0.01, ***P≤0.001) by one-way ANOVA followed by Bonferroni’s multiple comparison test (n = 3).

Next, we examined PAR_1_-activated G_12/13_ signaling by measuring RhoA activation after cell stimulation with either thrombin or PAR_1_-AP. In Met-5A cells, 10 nM thrombin induced a significant 2.5-fold increase of RhoA activation while in NCI-H28 cells the increase was just 1.2-fold (Figure 5.A). The selective PAR_1_-AP (10 µM) was less effective in stimulating RhoA activation than thrombin in Met-5A cells but it still caused a significant increase (Figure 5.B). Similarly to thrombin, PAR_1_-AP induced a modest increase of RhoA activation in NCI-H28 cells (Figure 5.B). We also examined the expression levels of Gα_12_, Gα_13_, and RhoA in both cell lines by immunoblot analysis (Figure S1). Our results indicate Gα_12_ and RhoA expression levels were similar in Met-5A and NCI-H28 cells while Gα_13_ expression was significantly increased in NCI-H28 cells compared to Met-5A cells (Figure S1).

**Figure 6:**
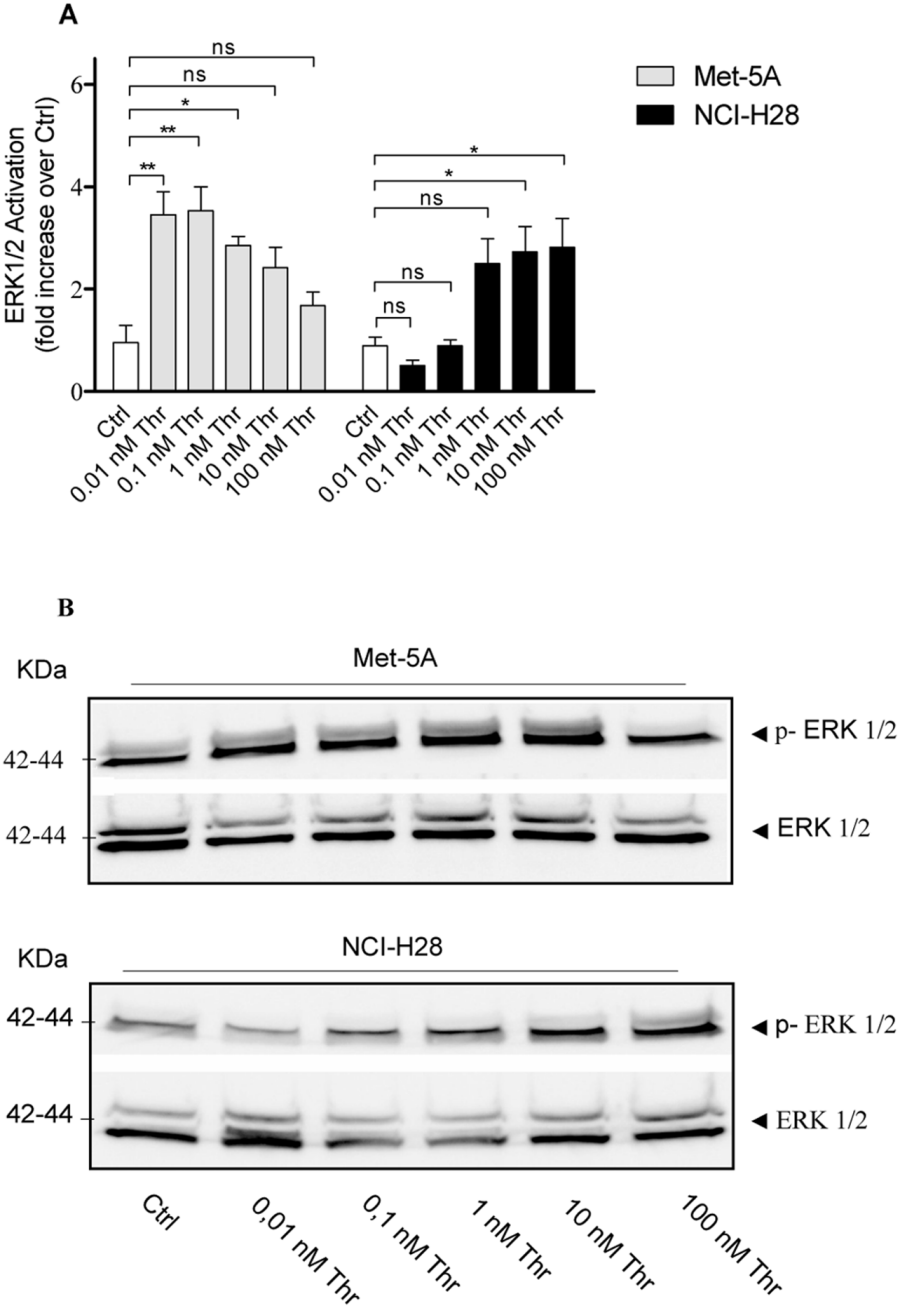
Thrombin differently induces ERK1/2 activation in Met-5A and NCI-H28 cells. A, relative intensity of pERK1/2 immunoreactive bands quantified by densitometric scanning. Serum and growth factor starved Met-5A and NCI-H28 cells were incubated in the presence and absence of various thrombin concentrations ranging from 0.01 to 100 nM for 5 min. ERK1/2 activation was then determined using a specific anti-phospho-ERK1/2 antibody. Nitrocellulose membranes were then stripped and reprobed for total ERK1/2. Data (mean ± SEM) are expressed as fold-increase over Ctrl and are the averages of three independent experiments performed in duplicate. The differences in phosphorylated ERK1/2 level between Ctrl (vehicle treated Met-5A or NCI-H28 cells) and thrombin-treated cells were significant (*P≤0.05, **P≤0.01) by one-way ANOVA followed by Bonferroni’s multiple comparison test. B, a representative immunoblot.

To further investigate distinctions in signaling, we examined thrombin induced ERK1/2 activation, an important mitogenic signaling cascade, in Met-5A and NCI-H28 cells. Thrombin (10 nM) caused a rapid increase of phosphorylated-ERK1/2 (pERK1/2) which reached a maximum level at 5 min and persisted up to 30 min in both cell lines (data not shown). Using a single time point (5 min) we examined the effect of various thrombin concentrations ranging from 0.01 to 100 nM and found that a maximal response was induced by 0.1 nM thrombin in Met-5A cells while higher thrombin concentrations reduced pERK1/2 (Figure 6). In contrast, NCI-H28 cells demonstrated maximal pERK1/2 activity at 10 nM thrombin (Figure 6). Of note, PAR_1_-induced ERK1/2 phosphorylation patterns in Met-5A and NCI-H28 cells were quite similar to respective thrombin-induced cell proliferation profiles (Figure 2.A).

### Prevalent intracellular PAR_1_ localization in NCI-H28 cells

**Figure 7:**
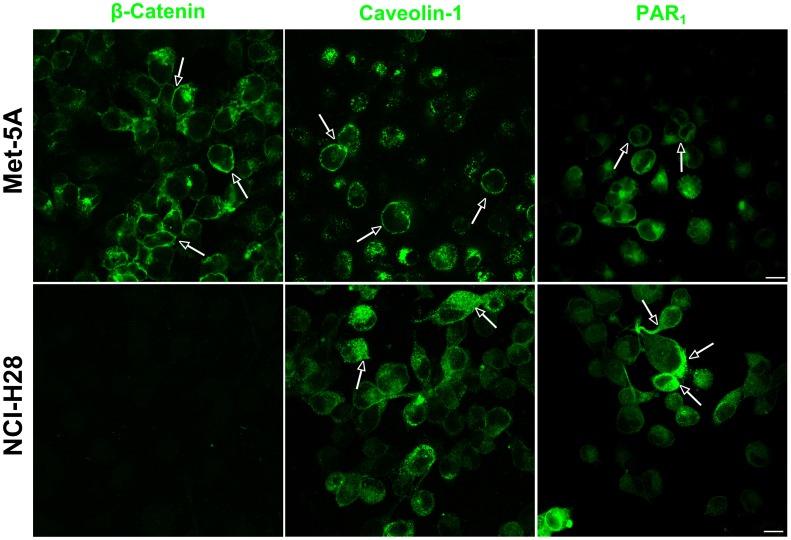
Cellular distribution of caveolin-1 and PAR_1_ in Met-5A and NCI-H28 cells. Immunolabeling of β-catenin, caveolin-1 and PAR_1_ in Met-5A and NCI-H28 cells was performed as described in Materials and Methods. The images shown are representative of many cells examined in two independent experiments. The arrows point out intracellular or plasma membrane localization of immunostained proteins. Scale Bar: 10 µm.

**Figure 8:**
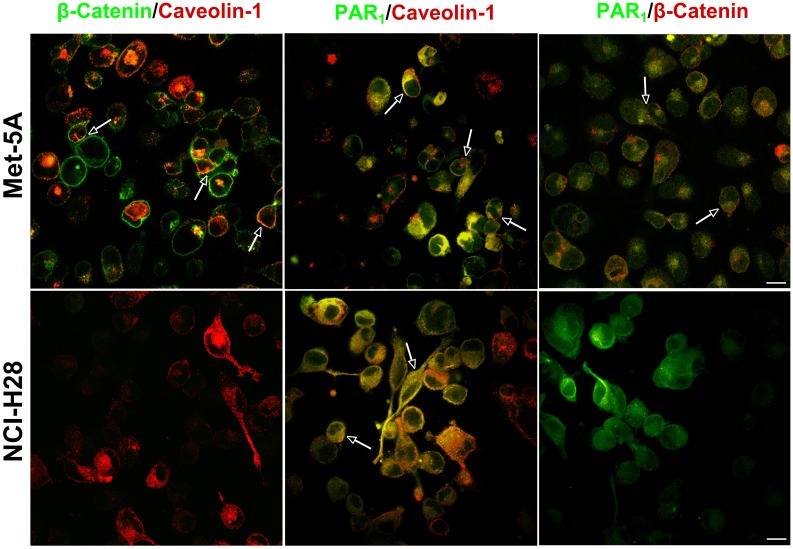
Double immunofluorescence labelling of caveolin-1 and PAR_1_ in Met-5A and NCI-H28 cells. Double labelling was performed by incubating antibodies as follow: anti-PAR_1_ and anti-caveolin-1; anti-PAR_1_ and rabbit polyclonal anti-β-catenin; mouse monoclonal anti-β-catenin and anti-caveolin-1. To visualize double fluorescent staining, cells were incubated with Alexa Fluor 488- and Alexa Fluor 568-labeled goat anti-mouse and anti-rabbit antibodies as described in Materials and Methods. The images shown are representative of many cells examined in two independent experiments. The yellow stain indicates protein proximity (see arrows). All images were analyzed using the ImageJ program [34]. PCC values are expressed as mean ± SEM of six examined area. PCC value for PAR_1_/caveolin-1 colocalization was 0.77±0.05 and 0.84±0.03 in Met-5A and NCI-H28 cells, respectively. PCC values for PAR_1_/β-catenin and caveolin-1/β-catenin colocalization in Met-5A cells were 0.70±0.02 and 0.55±0.04, respectively. Scale Bar: 10 µm.

In human umbilical vein endothelial cells, it has been reported that β-catenin greatly facilitates recruitment of caveolin-1 to VE-cadherin/catenin complex at cell junctions [44]. Additionally, several lines of evidence indicate that caveolae are relevant for GPCRs/G proteins signaling including that driven by PAR_1_ [45], [46]. As NCI-H28 cells have a homozygous deletion of the β-catenin gene we questioned whether the lack of this protein could reduce cell membrane recruitment of both caveolin-1 and PAR_1_. Therefore, we analyzed β-catenin, caveolin-1 and PAR_1_ localization in Met-5A and NCI-H28 cells by immunocytochemistry (Figure 7 and 8). In Met-5A cells, both β-catenin and caveolin-1 were localized on the plasma membrane including at some cell junctions and PAR_1_ also showed a prevalent but not exclusive localization on the plasma membrane (Figure 7). In contrast, in NCI-H28 cells there was no β-catenin staining, and caveolin-1 and PAR_1_ were mainly localized in the cytoplasm (Figure 7). In Met-5A cells, double labeling studies suggested β-catenin and caveolin-1 closely localized at cell junctions. In addition, both intracellular and plasma membrane PAR_1_ apparently colocalized with caveolin-1 (Figure 8). In NCI-H28 cells, the intracellular PAR_1_ was also in close proximity to caveolin-1 as suggested by the yellow stain (Figure 8). A quantification of PAR_1_/caveolin-1 colocalization using Pearson’s correlation coefficient (PCC) indicated a good degree of correlation in both Met-5A (PCC = 0.77±0.05; n = 6) and NCI-H28 cells (PCC = 0.84±0.03; n = 6).

### Neither β-catenin rescue nor deletion affect cell surface PAR_1_ expression

**Figure 9:**
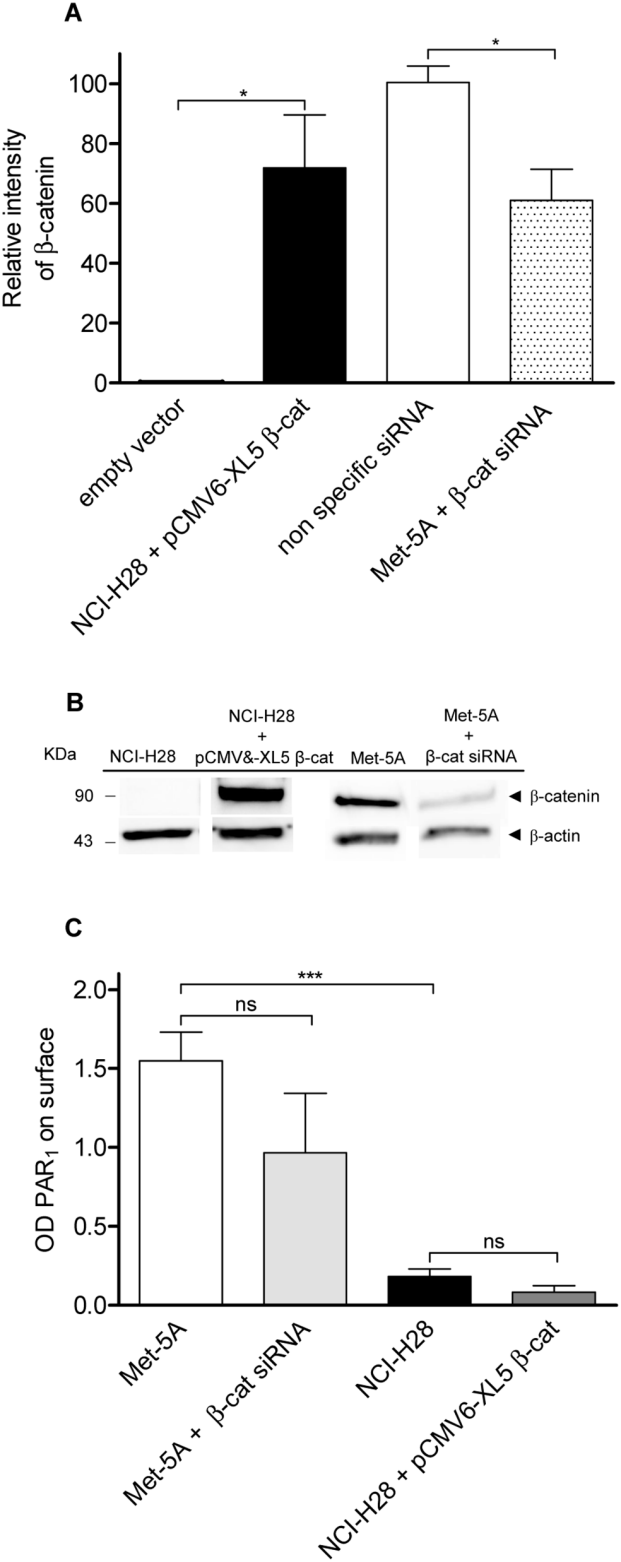
Neither β-catenin rescue nor deletion modify cell surface PAR_1_ expression. NCI-H28 cells were transiently transfected with plasmide vector containing CTNNB1 or empty vector (Ctrl) while Met-5A cells were transfected with nonspecific (Ctrls) or specific β-catenin siRNA as described in Materials and Methods. A, relative expression levels of β-catenin. Transfected cells were lysed and total cell proteins were analysed by immunoblot using an anti-β-catenin antibody. Then membranes were reprobed with an anti-β-actin antibody. Data are expressed as arbitrary unit (fold variation over Ctrl) after normalization by β-actin. Data shown are mean ± SEM of three independent experiments. The differences of β-catenin relative levels between Ctrls and cell transfected with the recombinant vector or specific siRNA were significant (*P≤0.05) by one-way ANOVA followed by Bonferroni’s multiple comparison test (n = 3). B, a representative immunoblot. C, cell surface PAR_1_ expression measured by ELISA assay. Antibody binding to fixed transfected cells was detected by horseradish peroxidise-conjugated secondary antibody. Data represent the mean ± SEM of three independent experiments performed in triplicate. The differences in cell surface PAR_1_ expression between Ctrls and cell transfected with the recombinant vector or specific siRNA were significant (***P≤0.001) by one-way ANOVA followed by Bonferroni’s multiple comparison test (n = 3).

In order to test our hypothesis that β-catenin is required for proper cell surface PAR_1_ localization, we transiently transfected NCI-H28 cells with a plasmide vector containing human β-catenin cDNA and silenced β-catenin expression in Met-5A cells using a specific siRNA. Immunoblot analysis indicated that in NCI-H28 cells transfected with the recombinant vector, β-catenin was expressed at high levels (Figure 9.A and 9.B) compared to the expression level in cells transfected with the empty vector. On the other hand, we also obtained a consistent reduction of β-catenin expression in Met-5A cells transfected with the β-catenin siRNA as compared to cells treated with a nonspecific scrambled siRNA (Figure 9.A and 9.B). However, in ELISA assays β-catenin transfected NCI-H28 cells did not show any increase of cell surface PAR_1_ expression while silenced Met-5A cells had no significant decrease of cell surface receptor as compared to control cells (Figure 9.C). Using immunofluorescence microscopy, we were also unable to detected any important change of PAR_1_ localization in β-catenin transfected and silenced cells as compored to respective controls (data not shown). All together, our findings indicate that the lack of β-catenin is not responsible for reduced cell surface PAR_1_ localization.

## Discussion

Coagulant proteases and PARs have been implicated in several types of malignant tumors. Indeed, a well-documented link between hyperactivation of the coagulation cascade and tumor progression exists. The pro-coagulant activity mediated by the action of coagulant proteases such as thrombin can contribute to the malignant phenotype both directly, by stimulating tumor cell proliferation, and indirectly through the development of tumor-associated thromboemboli [47]. Among cancer patients, those with MPM are very susceptible to thromboembolic complications [48]. In addition, Keshava *et al.* [22] have shown that MPM cell lines, which express tissue factor and PAR_1_ generate large tumors in mouse thoracic cavity thus indicating that activation of PAR_1_ promotes MPM cell growth.

To this end we investigated whether a correlation exists between PAR_1_ expression and cell proliferation using a MPM cell line (NCI-H28) and a nonmalignant pleural mesothelial cell line (Met-5A). In the NCI-H28 cell line, thrombomodulin, a transmembrane glycoprotein that controls thrombin-mediated proteolysis, is silenced by an epigenetic mechanism [24]. We found that the proliferative response of NCI-H28 cells to various thrombin concentrations was quite different from that obtained with the nonmalignant pleural mesothelial cell line. Whereas in NCI-H28 cells, thrombin–induced proliferation increased in a concentration dependent fashion, in Met-5A cells thrombin induced the maximal effect at 1 nM and then at higher concentrations the stimulatory effect progressively decreased. The proliferative response of NCI-H28 cells increased without reaching any growth steady state as expected when cells lose contact inhibition, a typical characteristic of cancer cells. The diverse response can result as consequence of reduced cell surface localization of PAR_1_ (Figure 3) in NCI-H28 cells even though the total receptor amount is increased. However, we do not feel to exclude that the lack of thrombomodulin in NCI-H28 cells [24] affects PAR_1_ growth signaling.

The non-selective PAR_1_-AP, SFLLRN-NH_2_, enhanced proliferation of both nonmalignant pleural mesothelial and MPM cells in a concentration-dependent fashion [13]. However, the proliferative response was slightly less marked than that observed with thrombin suggesting that either thrombin is also acting through other receptors or PAR_1_ activation by proteolytic cleavage elicits a cellular response which is not completely identical to that induced by a “free” synthetic peptide agonist. Backhart *et al.* [49] have reported that distinct cellular responses can be evoked by thrombin versus synthetic peptide agonists. In addition, McLaughlin *et al.* [50] have demonstrated that thrombin-activated PAR_1_ preferentially couples to G_12/13_ proteins while PAR_1_-APs favor activation of G_q_ signaling leading to [Ca^2+^]_i_ increase. The modest enhance of cell proliferation induced by the selective PAR_1_-AP suggests that PAR_2_ may also contribute to thrombin- and SFLLRN-NH_2-_stimulated functional response in both cell lines. Although thrombin is not able to cleave and activate PAR_2_, thrombin-cleaved PAR_1_ can transactivate PAR_2_ in human umbilical vein endothelial cells [51]. Indeed, as mentioned before, we were able to detect similar levels of PAR_2_ expression in Met-5A and NCI-H28 cells (data not shown).

When PAR_1_-mediated activation of signaling pathways was examined, we immediately noticed that G_q_ and G_12/13_ signaling was compromised in NCI-H28 cells. In this MPM cell line, the only signaling pathway which was fully activated by thrombin-cleaved PAR_1_ is through G_i_ proteins leading to inhibition of adenylyl cyclase. Indeed, thrombin inhibited cAMP production in a concentration-dependent fashion in NCI-H28 cells while in Met-5A cells it showed a biphasic effect. Simultaneous activation of different G proteins with release of a plethora of Gβγ subunits which are able to activate some isoforms of adenylyl cyclase can be responsible for the biphasic shape of the curve [52]. It is interesting to note that the selective PAR_1_-AP did not cause any major inhibition of cAMP accumulation. These findings are in agreement with thrombin and PAR_1_-AP displaying functional selectivity at PAR_1_ as reported by McLaughlin *et al.* [50].

Decreased G_q_ and G_12/13_ signaling with the prevalence of G_i_ signaling can explain the altered proliferative response to thrombin in NCI-H28 cells. Indeed, PAR_1_-mediated activation of ERK1/2 occurs through both G_q_ and G_i_ signaling with consequent activation of mitogenesis [53]. When we examined thrombin-induced ERK1/2 activation we found that lower thrombin concentrations were able to activate ERK1/2 in Met-5A than in NCI-H28 cells. This finding supports the role of G_q_ signaling in mediating thrombin-induced ERK1/2 activation in Met-5A. Persistent PAR_1_ signaling as consequence of altered receptor trafficking has been reported in metastatic breast carcinoma cells leading to enhanced cellular invasion [20]. We might speculate that altered PAR_1_ signaling can also impact MPM cell invasiveness.

Compartmentalization of PARs and G proteins in plasma membrane lipid raft microdomains such as caveolae can confer PAR/G protein selectivity [21]. Russo *et al.* [46] have shown the critical role of caveole in activated protein C (APC) activation of PAR_1_ selective signaling in endothelial cells. Furthermore, some studies concerning other GPCRs have demonstrated that caveolin-1 is required to prolong G_q_ signaling and inhibit receptor coupling to G_i/o_ proteins [54], [55]. In thrombin-stimulated endothelial cells, caveolin-1 opens cell junction by targeting catenins [44]. The recruitment of caveolin-1 at cell junctions is greatly facilitated by the presence of β-catenin in the cadherin/catenin complex. In NCI-H28 cells, a homozygous deletion of the β-catenin gene (*CTNNB1*) has been demonstrated suggesting that in these cells caveolin-1 is not completely associated to the plasma membrane [21]. Our immune fluorescence experiments show that in NCI-H28 cells caveolin-1 is partially retained in the cytoplasm while in Met-5A cells it is prevalently localized to the plasma membrane. In Met-5A cells, PAR_1_ is distributed in both plasma membrane and intracellular compartments and double immunolabeling studies suggest its proximity to caveolin-1. In NCI-H28 cells, PAR_1_ is mostly retained in the intracellular compartment. Of note, PAR_1_ and caveolin-1 appear to colocalize in both cell lines as suggested by PCC values. The intracellular retention of the receptor is confirmed by ELISA showing a consistent reduction of cell surface PAR_1_ in NCI-H28 cells compared to Met-5A cells. However, we do not know whether in NCI-H28 cells the increased intracellular receptor distribution is due to altered cell surface recruitment or enhanced-internalization of activated receptor. Of note, REN cells, another MPM cell line, which express similar PAR_1_ levels than Met-5A cells, also show a reduction of cell surface PAR_1_ by ELISA assay (see Figure 3). This aggressive MPM cell line does not express thrombomodulin as the NCI-H28 cell line and expresses high levels of tissue factor and very little amount of endothelial cell protein C receptor [22]. Thus, these evidences suggest that the observed reduction of cell surface PAR_1_ expression in these MPM cell lines can result as consequence of activated-receptor internalization. In order to exclude a role of β-catenin in recruiting PAR_1_ to the plasma membrane, we performed both rescue and deletion experiments and evaluated cell surface receptor expression by ELISA. However, our findings indicate that β-catenin expression is not required for cell surface PAR_1_ localization in both NCI-H28 and Met-5A cells. Since the NCI-H28 cell line is only one among other MPM cell lines examined, which shows a highly significant increase of PAR_1_ expression compared to Met-5A and human primary mesothelial cells, we may speculate that β-catenin indirectly modulates PAR_1_ expression at transcriptional level.

In summary, we have demonstrated that PAR_1_ is highly over-expressed in a MPM cell line, NCI-H28, while other three MPM cell lines show similar or slightly increased expression levels than a mesothelial cell line (Met-5A) and human primary mesothelial cells. Thrombin promotes Met-5A and NCI-H28 cells proliferation through activation of PAR_1_. In NCI-H28 cells, PAR_1_ although over-expressed, is defective in cell surface localization and signaling through G_q_ and G_12/13_ pathways. Cell surface PAR_1_ expression is also reduced in MPM REN cells, thus suggesting receptor activation and internalization by cell produced proteases in both cell lines. Further studies are needed to investigate the role of cell surface or secreted proteases in inducing PAR_1_ activation and stimulation of MPM growth.

## Supporting Information

Figure S1 **Expression of Gα subunits, RhoA, PLCβ_1_, and caveolin-1 in Met-5A and NCI-H28 cells.** Cells were lysed and protein solubilized as described under Materials and Methods. Proteins were then separated by SDS-PAGE and transferred onto nitrocellulose. Specific anti-Gα_q_, -Gα_12_, -G_13_, -RhoA, -PLCβ_1_, and -caveolin-1 antibodies were used to detect each protein. Nitrocellulose membranes were subsequently stripped and reprobed with an anti-β-actin antibody. The intensity of the immunoreactive bands was quantified by densitometric scanning. A, relative intensity of immunoreactive bands. Data are expressed as arbitrary unit (fold increase over Ctrl, Met-5A) after normalization by β-actin. Data shown are mean ± SEM of three independent experiments. The differences in protein expression between Met-5A and NCI-H28 cells were significant (*P≤0.05) by one-way ANOVA followed by Bonferroni’s multiple comparison test (n = 3). B, a representative immunoblot. Polyclonal anti-Gα antibodies were obtained from ABCAM (Cambridge, UK) while monoclonal anti-RhoA and polyclonal anti-PLCβ_1_ antibodies were from EMD Millipore Biosciences (Billerica, MA) and Thermo Fisher Scientific (Waltham, MA), respectively.
(TIF)
