# Supplementary figures and images for: Altered Protease–Activated Receptor-1 Expression and Signaling in a Malignant Pleural Mesothelioma Cell Line, NCI-H28, with Homozygous Deletion of the β-Catenin Gene
Source: PLoS One. 2014 Nov 3;9(11):e111550. doi: 10.1371/journal.pone.0111550 (PMC4218765; doi:10.1371/journal.pone.0111550)

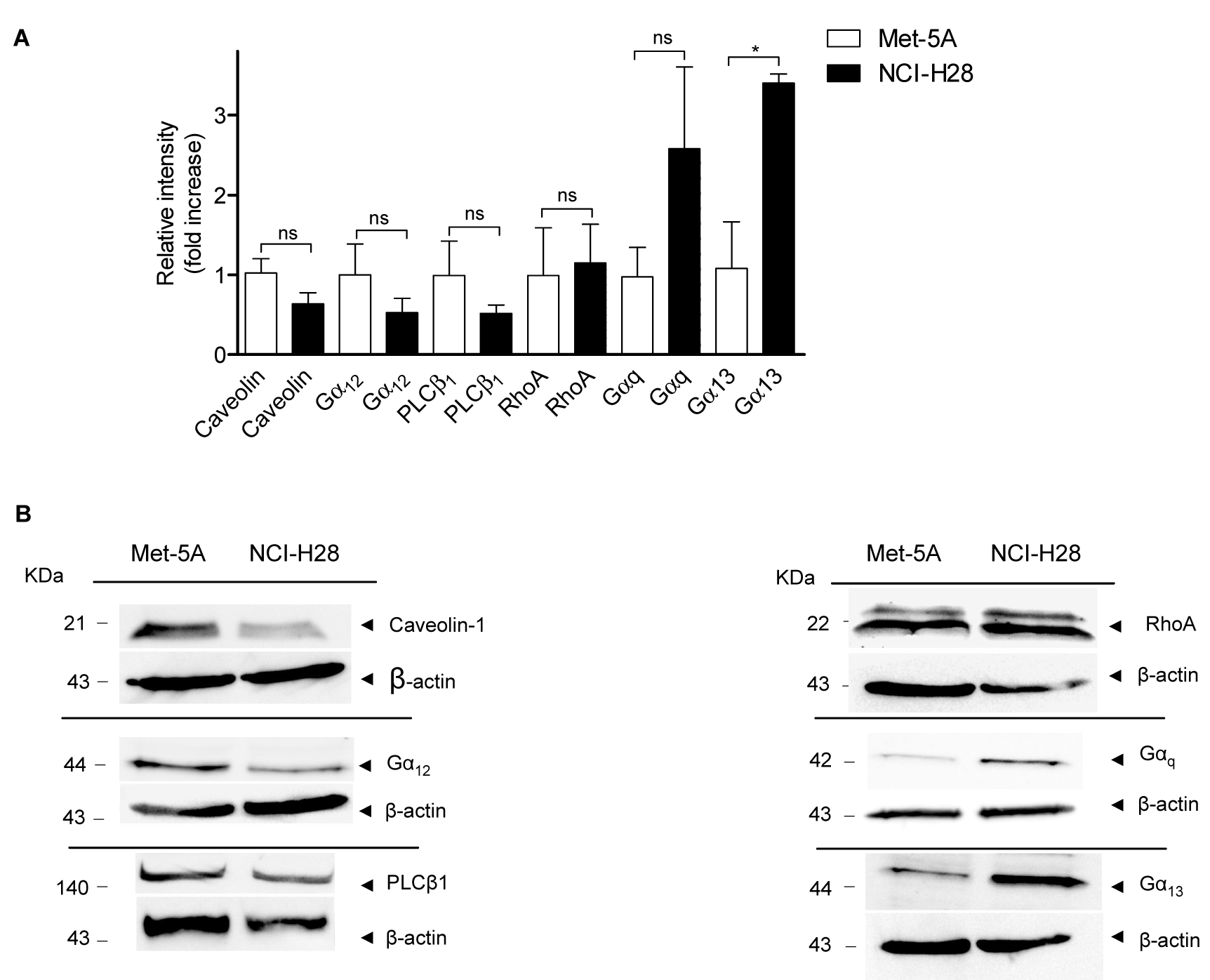

Supplement: Figure S1 — Expression of Gα subunits, RhoA, PLCβ1, and caveolin-1 in Met-5A and NCI-H28 cells. Cells were lysed and protein solubilized as described under Materials and Methods. Proteins were then separated by SDS-PAGE and transferred onto nitrocellulose. Specific anti-Gαq, -Gα12, -G13, -RhoA, -PLCβ1, and -caveolin-1 antibodies were used to detect each protein. Nitrocellulose membranes were subsequently stripped and reprobed with an anti-β-actin antibody. The intensity of the immunoreactive bands was quantified by densitometric scanning. A, relative intensity of immunoreactive bands. Data are expressed as arbitrary unit (fold increase over Ctrl, Met-5A) after normalization by β-actin. Data shown are mean ± SEM of three independent experiments. The differences in protein expression between Met-5A and NCI-H28 cells were significant (*P≤0.05) by one-way ANOVA followed by Bonferroni’s multiple comparison test (n = 3). B, a representative immunoblot. Polyclonal anti-Gα antibodies were obtained from ABCAM (Cambridge, UK) while monoclonal anti-RhoA and polyclonal anti-PLCβ1 antibodies were from EMD Millipore Biosciences (Billerica, MA) and Thermo Fisher Scientific (Waltham, MA), respectively. (TIF) [file pone.0111550.s001.tif]
